# Detection of *Chlamydia *in the peripheral blood cells of normal donors using *in vitro *culture, immunofluorescence microscopy and flow cytometry techniques

**DOI:** 10.1186/1471-2334-6-23

**Published:** 2006-02-10

**Authors:** Frances Cirino, Wilmore C Webley, Corrie West, Nancy L Croteau, Chester Andrzejewski, Elizabeth S Stuart

**Affiliations:** 1 Department of Microbiology, University of Massachusetts, Amherst Massachusetts, 01003, USA; 2 Department of Transfusion Medicine Baystate Medical Center, Springfield, Massachusetts, 01199, USA

## Abstract

**Background:**

*Chlamydia trachomatis *(*Ct*) and *Chlamydia pneumoniae *(*Cp*) are medically significant infectious agents associated with various chronic human pathologies. Nevertheless, specific roles in disease progression or initiation are incompletely defined. Both pathogens infect established cell lines *in vitro *and polymerase chain reaction (PCR) has detected *Chlamydia *DNA in various clinical specimens as well as in normal donor peripheral blood monocytes (PBMC). However, *Chlamydia *infection of other blood cell types, quantification of *Chlamydia *infected cells in peripheral blood and transmission of this infection *in vitro *have not been examined.

**Methods:**

*Cp *specific titers were assessed for sera from 459 normal human donor blood (NBD) samples. Isolated white blood cells (WBC) were assayed by *in vitro *culture to evaluate infection transmission of blood cell borne chlamydiae. Smears of fresh blood samples (FB) were dual immunostained for microscopic identification of *Chlamydia*-infected cell types and aliquots also assessed using Flow Cytometry (FC).

**Results:**

ELISA demonstrated that 219 (47.7%) of the NBD samples exhibit elevated anti-*Cp *antibody titers. Imunofluorescence microscopy of smears demonstrated 113 (24.6%) of samples contained intracellular *Chlamydia *and monoclonals to specific CD markers showed that *in vivo *infection of neutrophil and eosinophil/basophil cells as well as monocytes occurs. *In vitro *culture established WBCs of 114 (24.8%) of the NBD samples harbored infectious chlamydiae, clinically a potentially source of transmission, FC demonstrated both *Chlamydia *infected and uninfected cells can be readily identified and quantified.

**Conclusion:**

NBD can harbor infected neutrophils, eosinophil/basophils and monocytes. The chlamydiae are infectious *in vitro*, and both total, and cell type specific *Chlamydia *carriage is quantifiable by FC.

## Background

*Chlamydiae*, obligate intracellular bacterial pathogens, cause an array of medically and economically important infectious diseases. *Chlamydia trachomatis *(*Ct*), the most common cause of sexually transmitted bacterial disease, is also the world's leading cause of infectious blindness [1]. *Chlamydophila pneumoniae *(*Cp*) is a ubiquitous respiratory pathogen responsible for sinusitis, bronchitis, and 10–15% of community acquired pneumonia cases worldwide[2]. By age 20, ~50% of the population exhibits evidence of past infection by *C. pneumoniae *and re-infection is common throughout life [3]. *Cp *has attracted increasing interest because it is associated with an array of chronic human diseases that are not restricted to mucosal surfaces. *C. pneumoniae *has been implicated in the pathobiology of atherosclerosis [4-7] multiple sclerosis [8,9], Alzheimer's disease (AD) [10], reactive arthritis [11], and asthma [12].

Although *C. pneumoniae *has been implicated as a factor in this diverse array of chronic human diseases, it remains unknown whether it is the causative agent or is simply important in exacerbating these pathologies. By PCR, evidence of these organisms has been found in the peripheral blood mononuclear cells (PBMCs) of healthy blood donors, and in patients with coronary artery disease [13-16]. However, evidence of organism infectivity and information outlining the intra-host spread of either organism from an initial infection site in lung or genital tract to widely disseminated sites where pathology occurs remains to be fully defined. Using a rabbit model, and cell culture, studies have shown that alveolar macrophages serve as host cells for *Cp *[17], transporting it through the mucosal barrier to the lymphatic system, then beyond into the systemic circulation [18]. *Chlamydia *infected macrophages have been found in atherosclerotic plaques [19], and recent studies have revealed that *in vitro Cp *can infect human neutrophil granulocytes, and then initiate delays in their spontaneous apoptosis [20]. Evidence has been presented that *in vitro Cp *can infect B and T lymphocyte cell lines as well as human peripheral blood mononuclear cells and induce a cytokine response [21,22]. As demonstrated by PCR, *Ct *also disseminates within the host following experimental genital tract infection in a murine model, but the mechanisms involved have not yet been fully clarified [23-29].

We initiated an exploration of viable, infectious *Chlamydia *carriage in human blood cells. The current study was directed at assessing the prevalence of *Chlamydia *in samples from a Normal Blood Donor (NBD) population as well as identifying the different types of white blood cells that harbor this pathogen *in vivo *and quantifying their numbers by flow cytometry. Results from our study examining a random cohort of 459 normal donor samples indicated that (i) the average blood borne carriage rate of *Chlamydia *for this cohort is 24.6%, (ii) *Chlamydia *can be present in NBD peripheral blood granulocytic neutrophils and eosinophil/basophil cells, as well as monocytes, (iii) chlamydiae in 24.8% of NBD peripheral WBC are infectious as demonstrated by *in vitro *culture and (iv) FC can quantify both total infected cell load (ICL) and infected cell type specific load (ICSL) in the peripheral WBC population.

## Methods

### Sample collection

Approval for using residual material from routinely collected peripheral blood specimens from healthy normal blood donors was obtained from the Institutional Review Board at Baystate Medical Center. De-identified NBD residuals collected in ethylenediaminetetraacetic acid (EDTA) tubes were obtained from the Blood Bank at Baystate Medical Center in Springfield, MA from November 2001 to October 2002. Samples were collected monthly and stored at 4°C until tested. For other assessments, de-identified fresh blood sample residuals (FB), collected in EDTA tubes, were obtained on a weekly basis from patients seen at the University Health Services (UHS) and used immediately. Unless otherwise specified, conjugated secondary antibody reagents were obtained from Jackson ImmunoResearch Laboratories, West Grove PA; the standard incubation was 1 h at room temperature (RT) unless otherwise specified.

### *C. pneumoniae *specific peptide ELISA

A 14 amino acid *C. pneumoniae *specific peptide sequence deriving from the gene encoding the major outer membrane porin protein of *C. pneumoniae *AR 39 [30] was synthesized (Sigma Genosys, The Woodlands, TX). It served as the antigen in ELISA tests for *C. pneumoniae *specific antibody in NBD plasma samples. ELISA wells (NUNC™, Nunc A/S, Roskilde, Denmark) were coated for 1 h with 100 ul containing 1 μg/ml peptide in phosphate buffered saline pH 7.2 (PBS). Plasma samples were serially diluted in ELISA wells using 0.1% BSA in PBS and standard protocols for blocking (3% bovine serum albumin: BSA, Sigma, St. Louis, MO), incubations, and washes (X3 with 0.1%BSA/PBS). Detection of bound human antibody used alkaline phosphatase (AP)-conjugated goat anti-human IgG and after 45 min incubation and multiple rinses, 100 μl/well of P-Nitrophenyl Phosphate substrate (pNPP, Sigma, St. Louis, MO) was added. Absorbances at 405 nm were measured with a VERSAmax Microplate reader (Molecular Devices Sunny Vale, CA). Titers were determined by comparison of absorbance readings of known positive and negative controls with those of the test samples.

### Immunostain of normal blood donor smears

Smears prepared with ~20 μl/ slide of whole blood collected in EDTA were air dried then fixed 10 min in 70% cold methanol (MEOH). Following PBS rinses, slides were incubated in a moist chamber with a rabbit or guinea pig anti-*Chlamydia *EB serum (Biomeda Corp. Foster City, CA) then multiply rinsed in PBS. Bound anti-*Chlamydia *antibody was detected with fluoresein isothiocynate (FITC)-conjugated goat anti-rabbit or donkey anti-guinea pig IgG. Primary and secondary antibody incubations used the standard protocol noted above. After PBS rinsing X3, coverslips were mounted using Fluoromount G (BioWhittaker, Walkersville, MD), sealed, then examined and photographed using a Nikon Eclipse E600 epifluorescence microscope and a SPOT digital camera (Digital Instruments Inc.) or a Zeiss LSM 510 Meta Confocal System

### Culture of normal blood donor samples

The buffy coat (BC) containing WBC, was removed from each 5 ml NBD sample, rinsed X4 with sterile PBS, treated to release EBs and cultured on J774A.1 monolayers as described previously [30]. At 96 h post infection (pi) monolayers on coverslips were rinsed with PBS, fixed with 70% MEOH, rinsed again and immunostained with a rabbit anti-*Chlamydia *EB, and then mounted as noted above.

### Detection of *Chlamydia *infected Monocyte, Eosinophil/Basophil and Neutrophil

Smears of BC cell pellets from each 5 ml fresh blood samples (FB), average donor age 28 years, were fixed with 70% MEOH. Except as specified, immunostaining used rabbit polyclonal anti-*Chlamydia *EB antibody followed by a TRITC conjugated goat anti-rabbit antibody. Specific cell type detection used mouse monoclonals: anti-Human CD14 (0.2 μg/10^6^cells) (Sigma, Saint Louis, MO), or anti-Human CD16b (1.0 μg/10^6^cells) (BD Biosciences Pharmigen, San Diego, CA.). Binding was detected with FITC-conjugated goat anti-mouse IgG. For samples incubated with PE-conjugated anti-Human CDw125 (1.0 μg/10^6^cells), bound anti-*Chlamydia *antibody detection used FITC conjugated anti-rabbit antibody. Slides were incubated, rinsed, mounted and sealed as described above and digital images of optical sections through these samples were acquired with a Zeiss LSM 510 Meta Confocal System.

### Flow Cytometry assessment of a WBC dilution series

Flow cytometry (FC) quantification of *Chlamydia*-infected peripheral blood WBC used PBS rinsed BC cells from smear positive NBD samples. The WBC pool was fixed and permeabilized (1% paraformaldehyde and 1% Triton X-100, 10 min. at RT; Aldrich Chemical Company, Inc., Milwaukee, WI). PBS rinsed RBCs from the same samples were also pooled. BC and RBC were separately incubated with pre-diluted guinea pig anti-*Chlamydia *primary antibody followed by incubation with PE-conjugated F(ab')_2 _donkey anti-guinea pig IgG, PBS rinsed X3, and mono-dispersed using a nylon mesh filter (Lab-Line Instruments Inc, Melrose Park, IL). A WBC dilution series was prepared in separate tubes as follows: 100%, 90%, 80%, 70%, 60%, 50%, 40%, 30%, 20%, 10%, 5%, 2%, 1%, 0.5%, and 0% WBC/tube. Sufficient RBCs were added to obtain a total of 50,000 cells/tube and the series analyzed by FACScan (Becton, Dickinson and Company, Franklin Lakes, NJ).

### Flow Cytometry of FB WBCs dual stained for Monocytes, Eosinophil/Basophil and Neutrophils

BC isolated from FB samples was pre-screened for *Chlamydia *infected cells by anti-*Chlamydia *immunostaining as described above. BC from four smear positive samples and separately, BC from four smear negative samples were pooled. Each pool was washed, fixed and permeabilized as described above. After counting, aliquots of the cell pools were incubated with the polyclonal guinea pig anti-*Chlamydia *primary antibody and one of the three monoclonal antibodies, at the concentrations noted above. To detect *Chlamydia *and CD14 or CD16b, BC were incubated with PE-conjugated F(ab')_2 _donkey anti-guinea pig IgG and FITC-conjugated rabbit anti-mouse IgG. *Chlamydia *in BC immunostained with PE-conjugated anti-CDw125, were detected with FITC conjugated F(ab')_2 _donkey anti-guinea pig IgG. After standard incubations and rinsing, samples were mono-dispersed, counted, diluted to obtain 1 × 10^6 ^cells/ml of sample and analyzed using the FACScan instrument.

### Statistics

NBD sample data were analyzed using the SPSS^® ^11.5 graduate pack statistics program [SPSS, Inc., Chicago, IL]. Comparisons of culture and smear positivity used Cross-tabs with the Fisher exact test to uncover nonrandom associations between these two categorical variables and Chi-Squared test and Kappa measure of agreement to determine significance. To verify the reproducibility of culture and smear assessments all samples were initially tested in duplicate. Subsequently, approximately 20% of the samples were re-tested by each method. The SD and CV were calculated based on the differences in the positive and negative percentages determined by the original vs. the repeat tests. All tests were two sided, and the level of significance was p ≤ 0.05.

## Results

Preliminary observations of cells in blood smears of normal donor sample residuals and our studies of pediatric blood sample residuals [30] showed each could contain cells that were infected with *Chlamydia*. To screen normal donors for *Chlamydia *carriage in circulating PBMCs, and to assess infectivity, we utilized ELISA, immunofluorescence microscopy, *in vitro *culture and flow cytometry

### Presence of anti-*C. pneumoniae *antibody in NBD Samples

A random cohort of blood donor sample residuals was obtained from the Blood Bank unit at Baystate Medical Center over a 12 month period. Donor demographics are presented in Table 1. As shown, the average age of this cohort was 44 years and consisted of 55% male and 45% female donors. Plasma was removed from the samples, and an aliquot assayed by ELISA for anti-*C. pneumoniae *antibody. As Table 2 shows, 219 (47.7%) of 459 samples tested exhibited a positive titer against the 14 amino acid *C. pneumoniae*-specific peptide. This level of sero-positive status is fully consistent with published reports for this age group and indicates this cohort is not anomalous in this respect.

**Table 1:** Demographics: Random Blood Donor Cohort

**Average Donor Age**		44 years	
**Male**		252 (55%)	
**Female**		207 (45%)	
**Blood Type Distribution in Donor Cohort**			

**A**	**40%**	**B**	**10%**
**AB**	**4%**	**O**	**46%**

**Table 2:** *Chlamydia *Related Statistics: Normal Blood Donor Cohort

**Assessment**	**Positive**	**Negative**	**Total**
**Cp Serologic Status**	219 (47.7%)	240(52.3%)	459
**Smear Status**	113 (24.6%)	346(75.4%)	459
**Culture Status**	114 (24.8%)	345(75.2%)	459
**Culture and Smear Positive**	76 (67.3% of total Smear Positive)		N/A
**Culture and Smear Negative**	308 (89% of total Smear Negative)		N/A
**Culture Pos/Smear Negative**	38 (33.3% of total Culture positive)		N/A
**Culture Neg/Smear Positive**	37 (32.7%^-^of total Smear positive)		N/A

### Microscopic demonstration of peripheral blood cells infected by *Chlamydia*

**Figure 1:**
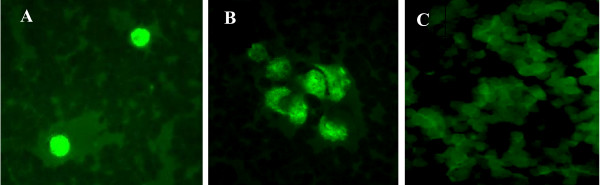
**Immunostained smears of NBD whole blood samples**. Smears were prepared by standard methods, immunostained using an anti-*Chlamydia *EB antibody and an FITC-conjugated secondary antibody. Epifluorescence images in **Panels A **and **B **show representative examples of *Chlamydia *positive smears exhibiting characteristic inclusion bodies. **Panel C **shows a representative smear negative sample and does not contain inclusion bodies or other *Chlamydia *positive material. Samples were photographed using a Nikon Eclipse E600 epifluorescence microscope and a SPOT digital camera. Original magnification 400×

Samples, including blood, are routinely assessed for *Chlamydia *by PCR and RT-PCR [5,13-15] but inherently PCR cannot demonstrate whether this DNA represents fragments or viable, infectious organisms [13-16] and immunostaining to detect infected PBMCs is not routinely used. This study therefore included immunofluorescent staining as described above to identify *Chlamydia*-infected NBD cells. As Figure 1A-C shows, *Chlamydia *positive material can be readily detected within lymphocyte-like and monocyte-like cells and examination of all 459 samples demonstrated 113 (24.6%) were positive for *Chlamydia *within peripheral WBCs (Table 2). The blood residuals, normally ≥30/month, were obtained over a 12 month period (Table 3) and comparison by month indicated that samples collected in December exhibited distinctly higher carriage of intracellular *Chlamydia*: 42% smear positive and 33% culture positive. In contrast, for August and November carriage was notably lower. Culture and smear positive results for these two months were 15%, 15% and 17%, 12% respectively. These divergent percentages may reflect a variable carriage rate in this cohort during the year since the sample numbers tested for each of these months were similar: 34, 35 and 39, respectively.

**Table 3:** Sample Distribution: Normal Donor Cohort Positive by Month

**Month 2001–2002**	**Sero-Positive**	**Smear Positive**	**Culture Positive**
November	50	17	12
December	97	42	43
February	63	22	28
March	24	20	22
April	55	30	30
May	37	31	24
June	40	27	24
July	70	24	30
August	10	15	15
September	33	24	27
October	70	20	25

### Demonstration *in vitro *of viable, infectious *Chlamydia *in WBC of peripheral blood samples

**Figure 2:**
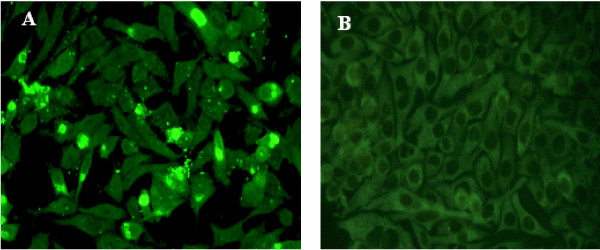
**Chlamydiae within Peripheral Blood Transfer Infection when Cultured *in vitro *on J774A.1 host cell monolayers**. BC from NBD peripheral blood samples were isolated, lysed and layered onto host cell monolayers as described. Epifluorescence images in **Panel A **show a representative example of a 96 h pi culture of a BC lysate from a *Chlamydia *smear positive sample and demonstrate these monolayers have remained healthy. **Panel B **shows a representative example of 96 h pi culture of a BC lysate from a *Chlamydia *negative smear sample. Note: panel A contains numerous cells with large inclusions, as well as cells with small inclusions and extracellular chlamydiae. These are characteristic of multiple replicative rounds in culture. Photographed using a Nikon Eclipse E600 epifluorescence microscope and a SPOT digital camera Original magnification 400×

Immunostain for *Chlamydia *antigens demonstrated their presence within PBMC. Therefore we next assessed whether infectious organisms per se are present. Chlamydiae are known to infect macrophages *in vivo *[17-19] and *in vitro *the J774A.1 macrophage monolayers are readily infected by *Chlamydia *and support growth for ~120 h pi. Using an effective culture method we developed previously and have described [30-33], WBC lysates were prepared and tested in duplicate on J774A.1 monolayers. At 96 h pi the monolayers were rinsed, fixed, and immunostained to detect *Chlamydia*. The results demonstrated that of the 459 samples, 114 (24.8%) were positive for growth of *Chlamydia *(Table 2). Figure 2 shows representative immunostained monolayers incubated with WBC derived inoculum from *Chlamydia *smear positive (Figure 2A) or negative samples (Figure 2B). At 96 h pi the cultures of smear positive samples contain large, late-stage inclusions as well as small, newly forming inclusions and released extracellular EBs. Table 2 shows that of the 459 samples tested 76 were positive by both smear and culture tests. Of the total positive samples, 37 were positive by smear but negative for cultivable organisms, potentially an indicator of persistence. Conversely 38 were negative by smear and positive by culture; this last finding may reflect the small sample size (20 μl) used in smear preparations. Together however the smear and *in vitro *culture findings demonstrated that not only do NBD samples carry PBMC infected by *Chlamydia *and visually identifiable by fluorescent microscopy, but frequently these WBC borne chlamydiae are infectious.

### Identification of *Chlamydia *infected Monocytes, Eosinophils/Basophils and Neutrophils in smears

**Figure 3:**
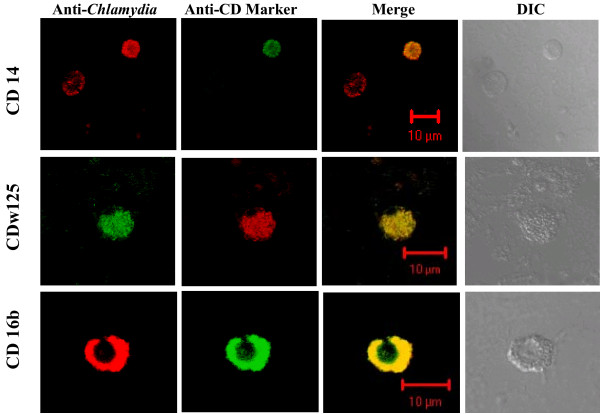
**Infected and uninfected WBCs dual immunostained with CD Specific monoclonal and anti-*Chlamydia *antibody**. Representative medial confocal microscopic images from the Z-axis series of optical sections through *Chlamydia*-infected human PBMC cells. Immunostaining used anti-*Chlamydia *EB antibody and either anti-CD14(monocyte), anti-CD 16b(neutrophil), or anti-CDw125 (eosinophil/basophil) mAb as indicated. Respectively, the Merge and DIC columns show the combined FITC and TRITC images, and the distribution and morphology of the cells in the field. Note: *In vivo *each of the three cell types can be infected by *Chlamydia *and the CD14 row includes a *Chlamydia *infected cell (TRITC positive) that is not immunostained by the ant-CD14 monoclonal (FITC negative).

**Figure 4:**
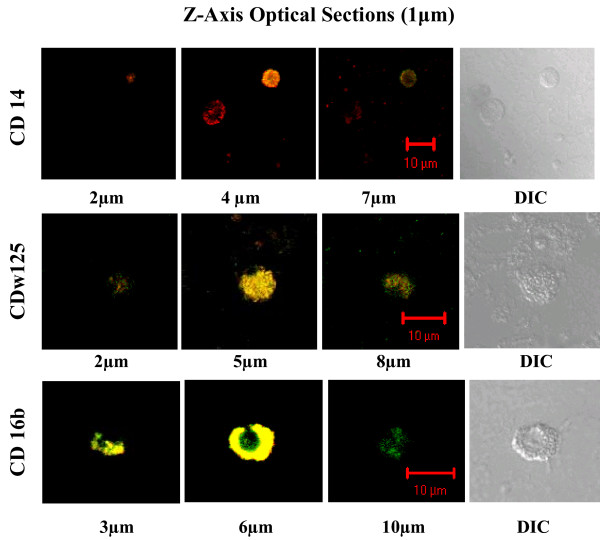
**Merged images at different depths from the confocal Z-axis series of dual immunostained WBCs**. These merged TRITC-FITC confocal images are from different depths in the same series of Z-axis optical sections acquired for each of the cell types shown in Figure 3. The position of individual sections relative to the cell surface is indicated in μm below each image. Optical section images were acquired with the Zeiss Metaconfocal System.

Despite the demonstration of *Chlamydia *DNA in PBMC samples, and demonstrations that *in vitro *numerous cell lines can be infected by different *Chlamydia *species and serovars, the specific types of WBC infected *in vivo *have not been fully examined. We selected three specific CD markers to identify WBC types and used them in conjunction with anti-*Chlamydia *antibody to immunostain WBCs in peripheral blood sample residuals. Since WBC inherently exhibit different life spans and can exhibit differential fragility, these assessments utilized fresh blood sample residuals. Smears of these FB samples were prepared, fixed and dual immunostained to detect *Chlamydia *and either CD14 (monocytes), CDw125 (eosinophils/basophils) or CD16b (neutrophil). Using Meta Confocal microscopy, optical sections through cells were examined and digital images acquired. Figure 3 shows representative examples of *Chlamydia*-infected monocyte (CD14), eosinophil/basophil (CDw125) and neutrophil (CD16b) cells. These can exhibit punctate fluorescent staining (row CD14 and CD125w) that reflects the expected uneven distribution of antigens. This is especially evident in non adherent cells and readily detectible in an optical section through a chlamydial inclusion. The anti-*Chlamydia *column shows the presence and distribution of *Chlamydia *positive material within the individual optical section, while the CD marker column identifies the cell type specific marker. The yellow or orange/yellow merge demonstrates the co-localization of the immunoprobes in that same optical section. The final column displays the DIC images and shows the position and general morphology of the individual cells. A comparison of the DIC and Merge images of the same row shows additional cells can be present that are not immunostained by either antibody (Figure 3-CD14 and CDw125). This observation serves to indicate the specificity of the immunoprobes. Figure 4 presents merged images obtained at three different levels from the same Z-axis optical sections through the cells shown in Figure 3. As the optical sections in the CD16b series show, the CD16b protein co-associates with the chlamydial antigens (6 μ) but also is present at the cell surface (10 μ).

### Flow Cytometry of serially diluted buffy coat pool

**Figure 5:**
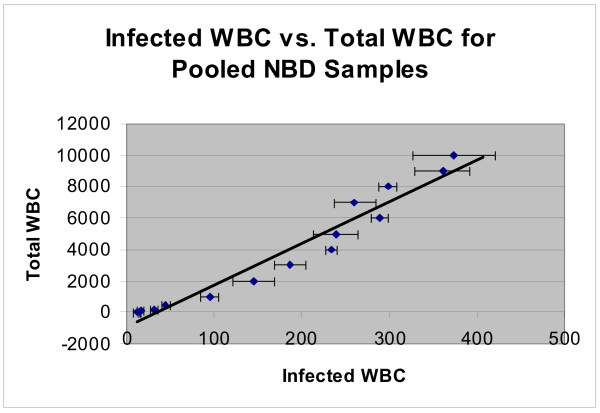
**FACScan Analysis of WBC Dilution Series from Donor Peripheral Blood Immunostained for *Chlamydia***. A buffy coat pool prepared from fresh human samples was assessed as positive for *Chlamydia *by immunostaining. The pool was immunostained for *Chlamydia*, serially diluted with RBCs pooled from the same samples then analyzed with a FACScan flow cytometer. The graphed results of the series demonstrates detection of *Chlamydia *infected cells in this non clinical down to 0.5% WBC/sample, a level of 3 ± 1.5 infected cells in the total of 10,000 cells counted.

Immunofluorescence allows detection of chlamydial antigens by microscopy but smears are inherently susceptible to significant sampling error. They also can not support quantification of total *Chlamydia *infected cell numbers in peripheral blood samples. We therefore utilized FC to quantify potentially 'sticky' *Chlamydia *infected BC samples. After pooling WBC pre-screened by immunostain for intracellular *Chlamydia*, a dilution series was prepared and FACScan assessments for each dilution analyzed (Figure 5). The data shows the series is linear over a broad range of infected cell numbers and that numeric differences between dilutions in the series are readily evident. As Figure 5 also shows, even in the most dilute sample that contains only 0.5% WBC, 3 ± 1.5 *Chlamydia *positive cells above background were detectible in the 10,000 cell sample. In addition to indicating the utility of FC for detecting *Chlamydia *in peripheral WBC samples, the series also serves as a control for subsequent FC directed towards quantifying specific WBC types infected by *Chlamydia in vivo*.

### Presence of *Chlamydia *in Monocytes, Eosinophils/Basophils and Neutrophils detected by Flow Cytometry

Given the demonstrated linearity and sensitivity, we utilized dual channel FC as a rapid, sensitive tool to quantify both the infected cell load (ICL) and the specific WBC types infected *in vivo*. Using fresh blood samples, two WBC pools were prepared and immunostained. One pool contained WBC from samples positive by blood smear anti-*Chlamydia *immunostain examination. The second pool, termed weakly positive, was prepared from samples *Chlamydia *negative by immunostain but positive by initial FC assessment. Aliquots of BC also were immunostained with a single antibody and used to establish the appropriate compensation for signal overlap between the two fluorescence channels and to establish appropriate, conservative demarcations of fluorescence positive.

**Figure 6:**
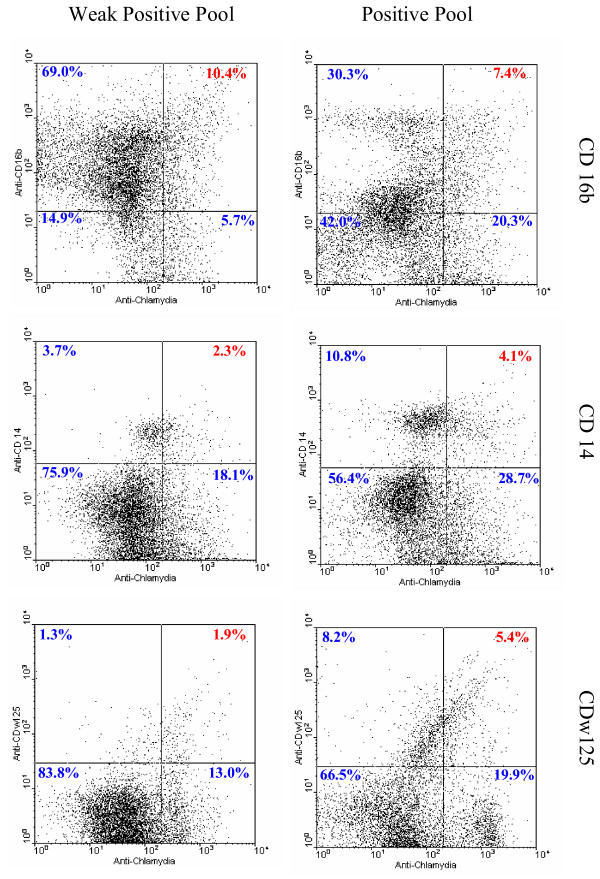
**Detection, identification and quantification of *Chlamydia *infected cells in buffy coat pools from FB samples using flow cytometry**. Fresh blood smears were assessed for *Chlamydia*-positive cells by immunostain. Aliquots of the pooled BC were dual immunostained to detect intracellular *Chlamydia *and identify CD16b, CD14, or CDSw125 cells. For each of the dual-immunostained BC pairs, aliquots of 10,000 cells were assessed. **Left panels: **results for the weakly positive pool. **Right panels: **results for the positive pool. The dot plots indicate: *in vivo *each cell type can be infected with *Chlamydia*, the different cell-types exhibit different patterns with different percentages of *Chlamydia*-infected cells and inherently the same preparation provides enumeration of uninfected as well as infected cells, thereby providing numerical totals for infection of each cell type/10,000 cells.

Peripheral WBC populations contain neutrophils (CD16b), monocytes (CD14) and eosinophil/basophils (CDw125) typically present at levels of 60–70%, 3–8%, and 1–4% respectively. Aliquots of each WBC pool were dual immunostained with anti-*Chlamydia *antibody and monoclonal antibody specific for CD16b, CD14, or CDw125 as described above. The dual channel FC data (Figure 6) indicate that in the population of 10,000 cells counted for the weakly positive pool, 10.4% were positive for both *Chlamydia *and the neutrophil marker, CD16b, while in the positive pool 7.4% exhibited dual positive fluorescence. Notably, the dot plot for the positive pool also contains a distinct distribution ('cloud') of cells lying somewhat above the CD16b line of demarcation that clearly is absent from the dot plot of the weakly positive sample. In this 'cloud' region, numerous cells in the positive sample exhibit a lower level of CD16b expression, as indicated by their location relative to the CD16b positive demarcation line. This low CD16b expression level would be consistent with the cells being immature neutrophils. Dual immunostaining of *Chlamydia*-positive monocytes (CD14) demonstrated that in the weakly positive pool, 2.3% of the 10,000 cells was dual positive, while in the positive pool, 4.1% of cells, nearly double, was dual positive. Finally, in aliquots immunostained to detect *Chlamydia *infected eosinophil/basophils (CDw125), dual positives were identified in 1.9% of cells (weakly positive sample) and 5.4% (positive sample), more than double the number of cells.

This quantitative dual channel FC data indicates that *in vivo *all three of these cell types can become infected, and is consistent with our identification in dual immunostained smears. The data also shows FC can quantify each cell type and that both the dot plot patterns and cell type specific numbers clearly differ for the weakly positive vs. positive samples. Therefore even at the minimal level of *Chlamydia*-infected cell carriage in these samples, cells of each type become infected *in vivo *and both the total infected cell load (ICL) and the infected cell type specific load (ICSL) can be quantified.

## Discussion

The studies presented here examined blood residuals obtained throughout the year and provide an assessment of blood cell borne *Chlamydia *carriage in a random cohort of normal blood donors. The aims were to determine the average rate of carriage, identify the key cell types infected *in vivo*, test whether *Chlamydia *infected cells can transmit infection to host cell monolayers, and assess quantification of the infected cell load in samples by using flow cytometry.

Our serological results demonstrated that in this donor population, 47.7% was sero-positive for *C. pneumoniae*. Given the average age of 44 years, this finding is consistent with the CDC report of serologic evidence of a previous infection by *C. pneumoniae *by the age of 20 (50%) [3] and demonstrates this donor cohort is not anomalous with respect to sero-positivity. Our assessments also showed a blood borne carriage rate for *Chlamydia*-infected cells of 24.6% positive. However, some notable differences were observed in the rate of *Chlamydia*-infected WBC, depending on the time of year (Table 3). Although only a multiyear study can verify seasonal variation, our finding suggests that the season of collection could potentially be a factor in the *Chlamydia *DNA detection rates of 18.5% to 46.15 % reported by others for PBMC. These PCR studies have not concomitantly tested for infectious organisms, nor is the time of year known [15,34-37]. Nevertheless, given published reports, the carriage rate we observed was not surprising, although the initial detection of blood borne *Chlamydia *carriage in immunofluorescent stained blood smears was unexpected. In addition, we also tested WBC from these samples for infectious organisms using an *in vitro *macrophage monolayer system, rather than the non-phagocytic HEp-2 or HeLa cells often used by others. As previously reported [30], this macrophage system is readily phagocytic and thus will take up the rare chlamydial EB in a more efficient manner. Moreover, using the J774A.1 cell monolayers and IMEMZO, we routinely cultured for 96 h pi, a time frame that supported re-infection within the monolayers. As Figure 2 shows, these cells continue to look healthy and the amplification achieved at 96 h pi allowed rapid identification of samples with infectious chlamydiae. Importantly, it also showed that the *Chlamydia *in these NBD cells can be infectious (Table 2, 24.8%).

The presence and especially the infectivity of blood cell borne chlamydiae is inherently relevant and potentially significant in medical contexts as diverse as tissue and organ transplantation [38,39], transfusion medicine and blood banking. As demonstrated previously by PCR [5,6,25,27] and here by immuno-detection and *in vitro *culture of intracellular organisms per se, a level of carriage clearly occurs. The demonstration that these organisms can be infectious suggests that removal of WBC from banked blood, for example, would be important and simple pre-storage leukoreduction is very effective [32]. In addition, where possible an assessment of the infected cell load in peripheral blood from an organ donor could be highly relevant to transplant surgery.

Our data also shows that not all samples positive for *Chlamydia *by examination of immunofluorescent smears were positive for infectious *Chlamydia *by *in vitro *culture (Table 2). This result may reflect the presence of persistently infected cells. *In vivo*, this pathogen can enter a persistent phase in response to various micro-environmental conditions. In this state it would be detected in immunofluorescent stained smears but produce few, if any infectious EBs. Although reversion from this state has not been directly demonstrated *in vivo*, studies have shown that *in vitro Chlamydia *can revert and give rise to infectious EBs [40-46]. Conversely, some immunostain negative samples were positive by culture. However, this result is not surprising and likely reflects the 20 μl sample size used for smear preparation vs. the much larger BC aliquot (50% of the total BC isolated from a 5 ml whole blood sample) used for the replicate *in vitro *cultures.

Recent experiments have shown *in vitro *infection of primary human neutrophils by *Chlamydia *as well as a concomitant delay of spontaneous apoptosis for infected neutrophils but not uninfected ones [20]. In contrast, the current study sought to identify blood borne WBC that had become infected by *Chlamydia in vivo*. Both immunofluorescence microscopy and flow cytometry demonstrate additional infective capababilities for this stealth pathogen. These studies used commercially available CD specific monoclonal antibody and both techniques demonstrated that non-clinical samples contain *in vivo *infected neutrophils and eosinophil/basophil cells as well as monocytes. PCR screening for *Chlamydia *of a portion of these samples verified *Chlamydia *DNA is present and in two instances where species specific primers were used, *C. trachomatis *and *C. pneumoniae *were present in the same sample (data not shown).

In terms of infected cell quantification, earlier studies had applied FC in various contexts relating to *Chlamydia *e.g. quantifying EB binding to standard host cell lines *in vitro*, establishing *Chlamydia *sensitivity to antibiotics or examining the role of estrogen receptors in *Chlamydia *infections [47-51]. However quantification of peripheral WBC infected *in vivo *by *Chlamydia *has not been reported, quite likely because infected cell carriage in non-clinical blood samples has been under appreciated. The FC quantification of *Chlamydia *infected WBC (Figure 5) demonstrated both sensitivity and linearity and provided a basis for the cell type specific quantifications comparing weakly positive and positive samples shown in Figure 6. For neutrophils (CD16b^+ ^cells) the FC data supports the suggestion that unusual numbers of putatively immature neutrophils may be present as a correlate of blood cell borne *Chlamydia *carriage. FC dot plots also demonstrated that the distribution patterns and the numbers of monocytes and eosinophil/basophil cells are distinctly different for these two pools. For the monocytes (CD14^+ ^cells), there were totals of 149,000 vs. 60,000 cells in the positive vs. weakly positive pool and the detected level of *Chlamydia *positive monocytes was 4.1% vs. 2.3%, a 1.8 fold difference. Interestingly, the total numbers of CDw125^+^, eosinophil/basophil cells/10,000 cells was much higher for the positive sample (1,360) vs. the weakly positive one (320), a 4.25 fold difference, as was the percentage of each pool that was infected (5.4% vs.1.9%), a 2.8 fold difference. After establishing parameters for routine use, a logical next step would be to assess WBC from patient samples and focus on clinical conditions in which a *Chlamydia *involvement is known or suspected.

Our evidence from the blood smear studies, as well as the earlier PCR data of others that indicated *Chlamydia *DNA carriage in normal populations, underscore the potential relevance of these and additional FC quantification studies. The demonstration that these chlamydiae can be infectious has implications for specific diagnoses, as well as health care in general. Clearly even low carriage levels of *Chlamydia*-infected cells can be quantified by FC (Figure 5) supporting determination of the total infected cell load in the peripheral circulation. Additionally, the FC dual immunostain data (Figure 6) shows that for specific cell types, both the total and infected cell populations are readily and simultaneously quantifiable. Such information would support establishing individual cell type specific load and potentially identify any skewing of infected cell types that might be associated with parameters such as levels of *Chlamydia*-infected WBC carriage or specific clinical presentations.

The significance of these specific findings and dot plot patterns remain to be established and will require additional broad based assessments. However, FC already is a routine diagnostic tool employed to enumerate CD4^+ ^T cells and CD34^+ ^progenitor cells and to screen for HLA-B27 antigen and leukemia/lymphoma immuno-phenotyping [52]. Thus once optimized for this application, FC could be routinely available to provide a non-invasive means of detecting and monitoring WBC infection by *Chlamydia *and could be of considerable diagnostic and prognostic value as well as a screening tool to for routine health assessments.

## Conclusion

Within the normal blood donor population tested, there is a *Chlamydia *carriage rate of 24.6%, contained in the WBC of the peripheral circulation. As demonstrated by fluorescent immunostaining combined with confocal microscopy and flow cytometric techniques, this pathogen is present within neutrophils and eosinophil/basophil cells, as well as in the monocyte/macrophage cells previously implicated. In addition, *in vitro *culture demonstrated *Chlamydia *in lysates of these non-clinical peripheral WBC can be infectious and readily transmits the *Chlamydia *to host cell monolayers. Therefore carriage of *Chlamydia *in the peripheral circulations could be significant in the context of transfusion or transplant recipients as well as routine health assessments.

## Competing interests

The author(s) declare that they have no competing interests.

## Authors' contributions

ESS supervised FC and NLC, directed the design and execution of the experiments and the draft manuscript and figures, and prepared the final version of the manuscript. WCW carried out the screening and statistical analysis of normal blood donor samples and the *in vitro *tests with isolated BC preparations to detect infectivity as a part of his doctoral work with ESS. WCW also provided training to NLC in the culture and *in vitro *infection of the cell lines, generation of pathogen stocks, and contributed to writing the final manuscript.

FC and CW carried out the flow cytometry, prepared and immunostained the smears of human peripheral blood cells to identify WBC types harboring intracellular *Chlamydia *and contributed to the manuscript preparation. FC carried out the confocal microscopy, image acquisition and figure preparation.

NLC carried out companion studies under controlled *in vitro *conditions and provided evidence supporting the observations of receptors initially detected in studies of cells from donor blood samples (manuscript in preparation).

CA coordinated NBD sample acquisition, provided expertise in Blood Banking and immunological related issues, as well as blood sample characterization, and provided valuable advice in manuscript preparation.

## Pre-publication history

The pre-publication history for this paper can be accessed here:


