# Correction: Detection of *Chlamydia *in the peripheral blood cells of normal donors using *in vitro *culture, immunofluorescence microscopy and flow cytometry techniques

**DOI:** 10.1186/1471-2334-6-165

**Published:** 2006-11-16

**Authors:** Frances Cirino, Wilmore C Webley, Corrie West, Nancy L Croteau, Chester Andrzejewski, Elizabeth S Stuart

**Affiliations:** 1 Department of Microbiology, University of Massachusetts, Amherst Massachusetts, 01003, USA; 2 Department of Transfusion Medicine Baystate Medical Center, Springfield, Massachusetts, 01199, USA

Upon close examination of the representative image depicted in 'figure 2' from [1] and a figure published elsewhere [2], we realized that the 'figure 2' of this work was a lower magnification of a section of the same microscope slide used in the previous manuscript [2]. The experiments leading to the data for these two manuscripts were generated during the same time period, in the same laboratory and were compiled, annotated and stored in the same folder, since pictures are taken in bulk at our microscopy facility on a fee for service basis. The figures both describe tissue culture of *Chlamydia *from clinical samples on the same tissue culture substrate (J774A.1 cells) and were in a single data base of figures with different alpha numeric designations.

We believe that sometime during the compilation and annotation process, the representative 'figure 2' previously shown in this work was incorrectly labeled. After careful scrutiny, we have determined that the image was in fact taken from a culture coverslip that was infected with lysates from pediatric bronchial lavage samples and not normal blood donor buffy coat (BC) samples. The original published figure was a 600× immunofluorescence image [2], part of which can be seen in the lower magnification image (400×) in this work.

The authors deeply regret this error in image annotation and later image selection and therefore submit a representative image from this work to replace the earlier incorrect image depicted in 'figure 2', which is provided with this manuscript (see figure 1).

**Figure 1:**
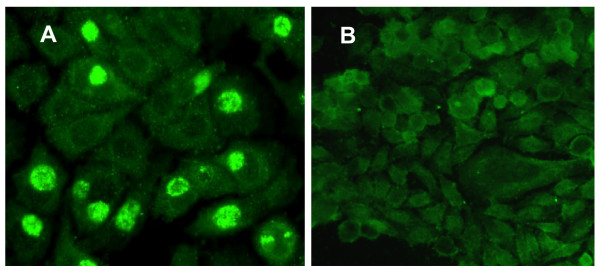
**This image is provided as a replacement for the original figure 2; Chlamydiae within Peripheral Blood Transfer Infection when Cultured *in vitro *on J774A.1 host cell monolayers**. BC from NBD peripheral blood samples were isolated, lysed and layered onto host cell monolayers as described. Epifluorescence image in **Panel A **shows a representative 96 h pi culture of a BC lysate from a *Chlamydia *smear positive sample. **Panel B **shows a representative example of a 96 h pi culture of a BC lysate from a *Chlamydia *negative smear sample. Note: panel A contains numerous cells with large inclusions, as well as cells with smaller inclusions. These are characteristic of multiple replicative rounds in culture. Photographed using a Nikon Eclipse E600 epifluorescence microscope and a SPOT digital camera Original magnification 400×.

## Pre-publication history

The pre-publication history for this paper can be accessed here:


